# *cyp51A* Mutations, Extrolite Profiles, and Antifungal Susceptibility in Clinical and Environmental Isolates of the Aspergillus viridinutans Species Complex

**DOI:** 10.1128/AAC.00632-19

**Published:** 2019-10-22

**Authors:** Jessica J. Talbot, Jens C. Frisvad, Jacques F. Meis, Ferry Hagen, Paul E. Verweij, David E. Hibbs, Felcia Lai, Paul W. Groundwater, Robert A. Samson, Sarah E. Kidd, Vanessa R. Barrs, Jos Houbraken

**Affiliations:** a Sydney School of Veterinary Science, Faculty of Science, The University of Sydney, Sydney, New South Wales, Australia; b Department of Biotechnology and Biomedicine, Technical University of Denmark, Kongens Lyngby, Denmark; c Department of Medical Microbiology and Infectious Diseases, Canisius Wilhelmina Hospital (CWZ), Nijmegen, the Netherlands; d Center of Expertise in Mycology Radboudumc/CWZ, Nijmegen, the Netherlands; e Westerdijk Fungal Biodiversity Institute, Utrecht, the Netherlands; f Department of Medical Microbiology, Radboud University Medical Center, Nijmegen, the Netherlands; g Faculty of Pharmacy, The University of Sydney, Sydney, New South Wales, Australia; h National Mycology Reference Centre, Microbiology and Infectious Diseases, SA Pathology, Adelaide, South Australia, Australia; i Marie Bashir Institute of Biosecurity and Infectious Diseases, The University of Sydney, Sydney, New South Wales, Australia

**Keywords:** cryptic species, *Aspergillus viridinutans*, *Aspergillus felis*, *Aspergillus udagawae*, azole resistance, *cyp51A*

## Abstract

The past decade has seen an increase in aspergillosis in humans and animals due to Aspergillus viridinutans species complex members. Azole resistance is common to these infections, carrying a poor prognosis. *cyp51A* gene mutations are the main cause of acquired azole resistance in Aspergillus fumigatus. This study aimed to determine if the azole-resistant phenotype in A. viridinutans complex members is associated with *cyp51A* mutations or extrolite profiles. The *cyp51A* gene of clinical and environmental isolates was amplified using novel primers, antifungal susceptibility was tested using the Clinical and Laboratory Standards Institute methodology, and extrolite profiling was performed using agar plug extraction. Very high azole MICs were detected in 84% of the isolates (31/37). The MICs of the newer antifungals luliconazole and olorofim (F901318) were low for all isolates. *cyp51A* sequences revealed 113 nonsynonymous mutations compared to the sequence of wild-type A. fumigatus. M172A/V and D255G, previously associated with A. fumigatus azole resistance, were common among all isolates but were not correlated with azole MICs. Two environmental isolates with nonsusceptibility to itraconazole and high MICs of voriconazole and isavuconazole harbored G138C, previously associated with azole-resistant A. fumigatus. Some novel mutations were identified only among isolates with high azole MICs. However, *cyp51A* homology modeling did not cause a significant protein structure change for these mutations. There was no correlation between extrolite patterns and susceptibility. For A. viridinutans complex isolates, *cyp51A* mutations and the extrolites that they produced were not major causes of antifungal resistance. Luliconazole and olorofim show promise for treating azole-resistant infections caused by these cryptic species.

## Abstract (precis)

The past decade has seen an increase in aspergillosis in humans and animals due to Aspergillus viridinutans species complex members. Azole resistance is common to these infections, carrying a poor prognosis. *cyp51A* gene mutations are the main cause of acquired azole resistance in Aspergillus fumigatus. This study aimed to determine if the azole-resistant phenotype in A. viridinutans complex members is associated with *cyp51A* mutations or extrolite profiles.

## INTRODUCTION

Aspergillosis, most commonly due to Aspergillus fumigatus, can cause invasive and fatal disease in humans and animals. Triazole antifungal drugs (itraconazole, voriconazole, posaconazole, and, more recently, isavuconazole) target fungal ergosterol synthesis and are the mainstay of treatment of *Aspergillus* diseases in humans and animals (1). Since the late 1990s, aspergillosis treatment in humans has been complicated by the development of azole resistance, most commonly reported in A. fumigatus isolates, leading to excess mortality in patients with azole-resistant invasive aspergillosis (2, 3). Additionally, poor clinical outcomes have been associated with infection by other species in *Aspergillus* section *Fumigati*, including members of the Aspergillus viridinutans species complex (AVSC), that demonstrate decreased antifungal susceptibility *in vitro* and *in vivo*, reports of which have increased (4).

Azole drug resistance can be due to innate or acquired mechanisms (5). Acquired resistance in A. fumigatus clinical and environmental isolates has most frequently been associated with tandem repeats in the promoter region and single point mutations of the *cyp51A* gene. In the past decade, acquired antifungal resistance was reported initially in human isolates from patients in the United States (6) and then in the Netherlands, where it was associated with the use of triazole fungicides in agriculture (7–9). Azole resistance due to *cyp51A* mutations has since been reported globally among clinical and environmental isolates (10), reinforcing the need to apply the World Health Organization concept of a one health approach in the management of fungal diseases (4, 11).

More than 50 mutations and tandem repeat combinations in the *cyp51A* gene have been detected among A. fumigatus isolates (12). Those most frequently associated with environmentally acquired azole resistance include TR_34_/L98H and TR_46_/Y121F/T289 (13), while a number of mutations have been associated with therapeutic exposure to azole drugs (G54, G138, G448, M220) (14).

The AVSC contains 10 species (A. arcoverdensis, A. aureolus, A. felis, A. frankstonensis, A. pseudoviridinutans, A. siamensis, A. udagawae, A. viridinutans, A. wyomingensis, A. acrensis) (15). In 2014, A. parafelis and A. pseudofelis were described as novel species closely related to A. felis; however, analysis of a larger number of strains indicates that they are actually A. felis (15, 16). Since 2005, four members of the AVSC have been increasingly associated with infections in humans and animals, including A. felis, A. pseudoviridinutans, A. udagawae, and A. wyomingensis (4). These species can cause localized or disseminated infections that are difficult to treat, with many isolates being noted to have high MICs of triazole antifungals (4). Innate resistance mechanisms are thought to be responsible for the high levels of intrinsic resistance encountered among these species (5). However, whether mutations of the *cyp51A* gene could be involved has not yet been investigated.

This study aimed to determine if there was any correlation between the azole-resistant phenotype and the *cyp51A* genotype among clinical and environmental AVSC isolates (see Table S1 in the supplemental material) through amplification of the *cyp51A* gene, antifungal susceptibility testing, and *cyp51A* protein homology modeling. An additional aim was to determine potential innate virulence factors of AVSC isolates by determining their extrolite production.

## RESULTS

### Antifungal susceptibility testing.

Of 37 AVSC isolates that sporulated adequately for antifungal susceptibility testing (see Table S2 in the supplemental material), 31 (83.8%) were defined as having high MICs of at least one azole, including itraconazole (20 of 37 [54.1%]; MIC, >1 μg/ml), voriconazole (31 of 37 [83.8%]; MIC, >1 μg/ml), isavuconazole (30 of 37 [81.1%]; MIC, >1 μg/ml), and posaconazole (1 of 37 [2.7%]; MIC, >0.5 μg/ml). Of these 37 isolates, 15 were of environmental origin, 14 were clinical isolates, and 2 were of unknown origin. A comparison of the geometric mean MIC, MIC ranges, MIC_50_, and MIC_90_ results for environmental versus clinical AVSC isolates is presented in Table 1. Environmental isolates had higher MICs than clinical isolates; however, this was not statistically significant (for isavuconazole, itraconazole, and posaconazole, *P* ≥ 0.2; for voriconazole, *P = *0.1 to 0.2). All isolates were inhibited by very low luliconazole and olorofim (F901318) concentrations (for luliconazole, the geometric mean MIC was 0.002 μg/ml, the MIC_90_ was 0.002 μg/ml, and the MIC range was 0.001 to 0.004 μg/ml; for olorofim, the geometric mean MIC was 0.008 μg/ml, the MIC_90_ was 0.016 μg/ml, and the MIC range was 0.002 to 0.016 μg/ml). Seven AVSC isolates (four clinical isolates, two environmental isolates, and one isolate of unknown origin) had low MICs for all azole drugs tested.

**TABLE 1:** Comparison of CLSI testing results for clinical versus environmental AVSC isolates^*a*^

Antimicrobial	MIC (μg/ml)							
	Range		Geometric mean		50%		90%	
	Clinical	Environmental	Clinical	Environmental	Clinical	Environmental	Clinical	Environmental
ITC	0.125 to >16	0.5 to >16	2.76	5.5	1	>16	>16	>16
POS	0.031 to 0.25	0.125 to 0.5	0.16	0.26	0.25	0.25	0.25	0.5
VRC	0.25 to 8	2 to 16	2.4	4.69	2	4	4	8
ISA	0.5 to 4	2 to 8	2.4	3.79	2	4	4	8
LUL	0.001 to 0.004	0.001 to 0.004	0.002	0.002	0.002	0.002	0.002	0.004
OLO	0.002 to 0.016	0.002 to 0.016	0.008	0.007	0.008	0.008	0.016	0.008

a Data are for 17 clinical AVSC isolates and environmental AVSC isolates. Data for three isolates with an unknown origin (DTO 019-D8, DTO 316-F9, and DTO 342-I3) were excluded from this table. Luliconazole data were available for 16/17 AVSC environmental origin isolates (no data were available for A. udagawae DTO 006-A3). Abbreviations: ITC, itraconazole; POS, posaconazole; VRC, voriconazole; ISA, isavuconazole; LUL, luliconazole; OLO, olorofim (F901318).

### *cyp51A* sequencing.

A total of 204 synonymous and 113 nonsynonymous mutations were identified among the 56 AVSC isolates (Table S3). Of the nonsynonymous mutations, all AVSC isolates tested, regardless of azole phenotype, had the mutations W6L, V15M, K80R, D255G, C270S, I367L, H403Y, and L464I. At amino acid position M172, all isolates had a mutation of either M172V (*n* = 55) or M172A (*n* = 1).

For isolates demonstrating high azole MICs, 15 unique mutations that were not present in isolates with low MICs were identified (Table 2). This included G138C, found in two environmental A. felis isolates that were nonsusceptible to itraconazole at the highest concentration tested and that had high MICs of voriconazole and isavuconazole. One of these isolates also demonstrated a posaconazole MIC at the A. fumigatus epidemiological cutoff value (ECV), and the other had an intermediate posaconazole MIC (Table 2). Both these isolates also harbored a unique single nucleotide polymorphism (SNP), T215S (Table 2). Two A. aureolus environmental isolates harbored the nonsynonymous mutation V101L; isolate DTO 278-B7 had a MIC at the A. fumigatus ECV only for posaconazole, while DTO 331-G6 did not sporulate to be used in antifungal susceptibility testing. Both these isolates also harbored unique *cyp51A* SNPs, A103T, A234V, I360V, V428I, Q423D, F478V, and G505R (Table 2).

**TABLE 2:** *cyp51A* mutations observed in high-azole-MIC-phenotype AVSC isolates that were not found in low-azole-MIC isolates^*a*^

Azole-resistant phenotype	Species (isolate no.)	*cyp51A* mutations
High multiazole MICs (ITC, ISA, VRC)	A. felis (DTO 341-E5)	T215S, G138C
High multiazole MICs (ITC, ISA, VRC)	A. felis (DTO 341-E4)	T215S, G138C
High multiazole MICs (ITC, ISA, VRC)	A. felis (DTO 131-E6)	S197C, Q340R
High-multiazole MICs (ITC, ISA, VRC)	A. viridinutans (DTO 050-F1)	A63S, L327P, V396A
Single azole MIC at the ECV for A. fumigatus (POS)	A. aureolus (DTO 278-B7)	A103T, V101L, A234V, I360V, V428I, G505R, Q423D, F478V

a MICs were determined by CLSI testing. See Table S3 in the supplemental material for a list of all mutations found. Abbreviations: ISA, isavuconazole; ITC, itraconazole; VRC, voriconazole; POS, posaconazole; ECV, epidemiological cutoff value.

**FIG 1:**
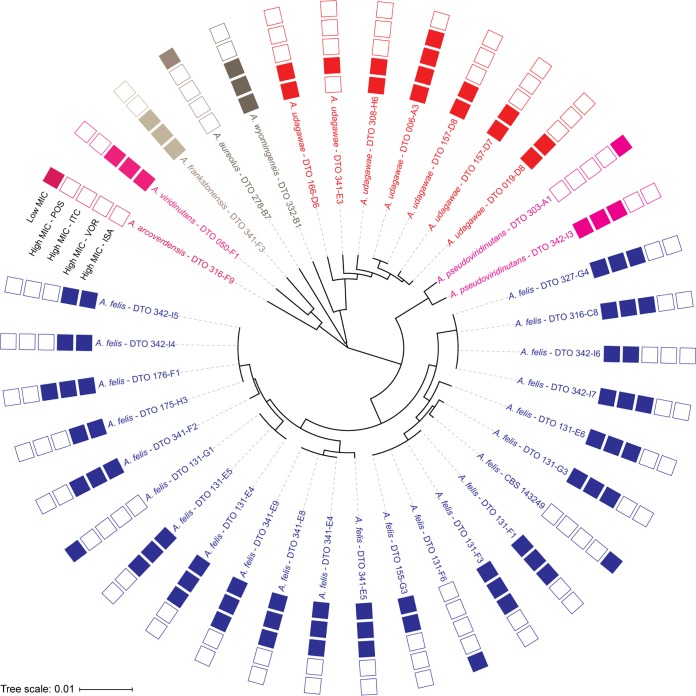
Phylogenetic tree of Aspergillus viridinutans species complex *cyp51A* sequences with corresponding low, high, or unknown triazole MICs. ISA, isavuconazole; ITC, itraconazole; VOR, voriconazole; POS, posaconazole.

A phylogenetic tree developed from the *cyp51A* sequences grouped isolates of the same species together (Fig. 1).

### *cyp51A* homology modeling.

Five AVSC *cyp51A* homology models (models F to J) were constructed to compare the amino acid profiles of isolates with low azole MIC values (model F, A. felis DTO 131-G1) to those of isolates with high azole MIC values (model G, A. viridinutans DTO 050-F1; model H, A. aureolus DTO 278-B7; model I, A. felis DTO 341-E5; model J, A. felis DTO 131-E6) (Fig. S1). Docking scores and molecular mechanics-generalized Born surface area (MMGBSA) analysis results are presented in Table 3. Compared to model F, there were no significant changes to the overall protein structure to confer resistance in models G to J. For itraconazole, the most negative docking score value was obtained with model I (−11.03 kcal/mol) and the most negative binding free energy [Δ*G*(binding)] value was with low-azole-MIC model F (−148.403 kcal/mol), with a higher potential for azole binding being seen in model F than in models G to J. For posaconazole’s interactions, the most negative docking score was obtained with model I (−11.54 kcal/mol) and the most negative Δ*G*(binding) value was obtained with model G (−148.061 kcal/mol). Conflicting results were found for voriconazole, with model H having the most negative docking score (−7.881 kcal/mol) and the Δ*G*(binding) value (−64.09 kcal/mol) having the highest potential for azole binding (Table 3). Root mean square deviation (RMSD) scores demonstrated high similarity among models G to J compared to the susceptible A. felis model (model F), with all models having a difference of less than 0.2 Å (model I = 0.2004 Å, model J = 0.1929 Å, model G = 0.0613 Å, model H = 0.0570 Å). Additionally, two A. fumigatus *cyp51A* homology models (models K and L) were constructed to determine if the mutations reported in association with azole resistance in A. fumigatus (positions G138 and V101) altered the protein structure in a way similar to that of the mutations in AVSC-based models H and I. This confirmed the results of AVSC models H and I, with no major difference for docking and MMGBSA analysis with these mutations in the A. fumigatus model (Table 3).

**TABLE 3:** Docking scores of the best conformers for posaconazole, itraconazole, and voriconazole and corresponding MMGBSA study values

Model^*a*^ (species)	Ligand^*b*^	Docking score (kcal/mol)	Ligand strain energy (kcal/mol)	Receptor strain energy (kcal/mol)	Δ*G*(binding) (kcal/mol)
F (A. felis)	Posaconazole 2	−9.549	20.174	43.264	−145.031
	Itraconazole 2	−10.348	10.148	61.584	−148.403
	Voriconazole	−7.284	2.101	18.284	−56.913
G (A. viridinutans*)*	Posaconazole 2	−9.457	17.582	20.2	−148.061
	Itraconazole 1	−7.972	11.809	48.485	−117.783
	Voriconazole	−7.13	2.618	23.192	−51.94
H (A. aureolus)	Posaconazole 2	−10.21	11.18	62.408	−117.291
	Itraconazole 4	−8.732	8.419	29.881	−134.629
	Voriconazole	−7.881	10.41	16.574	−64.09
I (A. felis; mutations T215S/G138C)	Posaconazole 2	−11.541	13.355	34.947	−140.137
	Itraconazole 1	−11.031	10.476	36.487	−122.160
	Voriconazole	−7.220	6.929	20.331	−50.546
J (A. felis; mutations S197C/Q340R)	Posaconazole 2	−10.275	16.779	36.947	−141.408
	Itraconazole 3	−10.318	14.646	36.402	−112.306
	Voriconazole	−7.461	6.916	24.457	−50.255
B (A. fumigatus)	Posaconazole 2	−10.385	11.936	34.031	−133.210
	Itraconazole 4	−9.543	11.503	10.276	−128.252
	Voriconazole	−6.991	2.048	−0.416	−49.793
K (A. fumigatus model of model I mutations)	Posaconazole 2	−9.859	12.880	30.784	−145.495
	Itraconazole 4	−9.002	11.412	52.439	−131.727
	Voriconazole	−7.570	11.101	−0.489	−72.311
L (A. fumigatus model of V101L/A103T/A234V/I360V/V428I/G505R/Q423D/F478V)	Posaconazole 1	−10.530	12.928	29.622	−122.594
	Itraconazole 2	−10.764	10.357	49.188	−148.968
	Voriconazole	−7.098	2.482	31.636	−49.211

a Models F to J are Aspergillus viridinutans species complex member-based models, and models B, K, and L are A. fumigatus-based models.

b Ligand column numbers proceeding antimicrobial name refer to the conformation number of the antimicrobial structure relative to ground state conformation, generated by the Ligprep program (Schrodinger release 2017-3). Only one conformation number was generated for voriconazole.

### Extrolite profiling.

Table S5 provides the extrolite profiles of the AVSC species tested. Aspergillus felis and A. udagawae species shared the production of only fumagillin and were otherwise chemotaxonomically different. Aspergillus felis generally produced a large number of extrolites, including antafumicins/clavatols, aszonalenins, cytochalasin E, fumagillin, helvolic acid, kotanins, and viriditoxin, while A. udagawae produced fumigatins, fumitremorgins, fumagillin, pseurotin A, tryptoquivalines, and tryptoquivalones.

## DISCUSSION

High azole MIC values were commonly detected for AVSC clinical and environmental isolates. These were most frequently for isavuconazole, followed by voriconazole and itraconazole, while high MICs of posaconazole were not common. These results are similar to those of a recent study of AVSC isolates, which also found high MICs of voriconazole and itraconazole and generally low MICs of posaconazole (17).

As patients with aspergillosis due to AVSC members commonly have poor clinical outcomes and high *in vitro* MICs of azoles, we tested their susceptibility to two newer antifungal drugs, luliconazole and olorofim (F901318). Luliconazole is a commercially available cytochrome P450 2C19 inhibitor imidazole antifungal. There are limited efficacy data for its use in the treatment of aspergillosis; however, *in vitro* antifungal susceptibility testing of A. fumigatus and A. terreus isolates and in *in vivo* animal models has demonstrated that it is a promising treatment alternative to currently used antifungals, has much lower MIC values than other antifungals (azoles, echinocandins, and polyenes), and results in improved survival rates (18–20). All AVSC isolates had luliconazole MICs in the same range reported for azole-susceptible A. fumigatus isolates (0.001 to 0.008 μg/ml) (19). The reference ranges were generally lower than those of the other azoles tested, consistent with the findings for other *Aspergillus* species (18, 20). Olorofim (F901318) is an orotomide antifungal drug that has proven *in vitro* activity against filamentous fungi with high MIC values of commonly used antifungal drugs or demonstrated azole resistance, including *Scedosporium*, *Lomentospora*, and *Aspergillus* species (21, 22). *In vivo* pharmacodynamics studies in animals with invasive pulmonary and sinopulmonary aspergillosis have demonstrated similar or improved survival outcomes in response to first-line triazole drugs, with similar survival rates reported for posaconazole-susceptible strains and improved survival reported in posaconazole-resistant strains, with pharmacodynamic targets being identified in phase 2 clinical trials (23–25). All our AVSC isolates had olorofim MICs below the range reported for azole-susceptible A. fumigatus isolates (0.031 to 0.125 μg/ml) (22). Although further evaluation is required, these antifungal drugs have promising potential as alternatives to the current azole therapeutics for *in vivo* therapeutic use against AVSC species.

Of the *cyp51A* SNPs identified only in isolates with high MICs, none were found to confer the same susceptibility phenotype (Table 2), so *cyp51A* modeling was used to further examine their significance, and the resulting homology models varied. Itraconazole interactions demonstrated the most consistent result, with model F having the strongest binding affinity to this drug compared to the rest of the models. This trend was also observed in posaconazole’s interactions, and voriconazole demonstrated conflicting results, which is most likely due to its smaller size, as it does not require much energy to position itself into the correct binding mode. These results and the similarity between the protein structures, as demonstrated via RMSD score calculations, suggest that the *cyp51A* mutations in these isolates do not cause a significant change in the overall protein structure and thus do not directly interfere with azole drug binding to confer resistance. Future work investigating the genome sequences of susceptible and resistant AVSC isolates could shed light on these amino acid profile differences, which have the potential to determine strain lineage as well as identify other genetic causes of azole resistance. Aspergillus felis is heterothallic, and progeny resulting from direct mating of susceptible and resistant A. felis isolates could be used in segregate analyses.

Although nonsynonymous mutations at sites previously associated with azole resistance in A. fumigatus were found (M172A, M172V, and D255G), these were present in AVSC isolates with both high and low azole MICs and so are unlikely to be a cause of azole resistance, as has also been reported for A. fumigatus wild-type (WT) isolates (26–40). However, the combination of single point mutations may also be important when determining the ability to cause azole resistance, as recently demonstrated by a study investigating A. fumigatus isolates harboring mutations at these positions (M172V and D255E) in combination with either F46Y, G89G, L358L, and C454C or F46Y, G89G, N248T, L358L, E427K, and C454C, which were shown to have elevated azole MICs and were pathogenic in Galleria mellonella host virulence studies and of a cluster/lineage different from strains of WT A. fumigatus (41). However, in our study this was not the case for isolates with high azole MIC values that underwent *cyp51A* homology modeling. Future studies could examine the transfer of mutated *cyp51A* genes that confer resistance from A. fumigatus to AVSC strains with low azole MICs and, similarly, the transfer of *cyp51A* genes from a high-azole-MIC AVSC strain to wild-type A. fumigatus to observe if an increase in azole resistance occurs. This could be achieved via gene editing, for example, by cloning or using the CRISPR/Cas9 system, as has been described previously for the *cyp51A* gene for *Aspergillus* species (42–45). Our study found the azole resistance-associated mutation G138C in two environmental A. felis isolates (DTO 341-E4, DTO 341-E5) from woodland soil in Australia. Interestingly, this mutation has previously been reported in A. fumigatus isolates of clinical origin and is thought to be acquired following therapeutic azole exposure and to confer resistance to itraconazole, voriconazole, posaconazole, and isavuconazole (40), similar to our phenotype results. However, this is not clear, as other single point mutations previously associated with in-host resistance development have also since been found in the environment (e.g., M220 and G54) (46, 47). Strains with these two mutations also harbored the T215S mutation, which has not previously been reported for A. fumigatus and which was not identified in any other isolates in this study. This mutation, in combination with G138C, may also contribute to acquired resistance; however, this was not supported by our *cyp51A* homology modeling of AVSC or A. fumigatus species. Additionally, it has previously been demonstrated via A. fumigatus *cyp51A* homology modeling that while G138C is closely positioned to the protein access channel opening, the mutation can actually increase stability for azole docking (48).

The mutation V101L was found in two A. aureolus isolates; however, only one of these sporulated adequately for antifungal susceptibility testing, demonstrating a posaconazole MIC at the A. fumigatus ECV. Mutations at this position in A. fumigatus have led to the change V101F, which, in association with M220I, confers multiazole resistance (itraconazole and posaconazole resistance with variable voriconazole resistance) (49). Mutations at M220 were not present in these or any other AVSC isolates. *cyp51A* homology modeling of these mutations in both the AVSC and A. fumigatus models did not confer changes consistent with azole resistance.

Nine *cyp51A* mutations present among all AVSC isolates (isolates with high and low MICs) compared to the sequence of the A. fumigatus WT (see Table S3 in the supplemental material) are likely nonfunctional in relation to azole-resistant phenotypes. Species-specific amino acid substitutions were also observed and could be interesting targets for identification purposes (at either the species or the complex level) or for the development of diagnostics, and the degenerate primers developed for this study have potential clinical application for other pathogenic, non- *fumigatus Aspergillus* species.

We found that isolates of A. felis, A. frankstonensis, A. pseudoviridinutans, A. udagawae, A. viridinutans, and A. wyomingensis were very competent in producing secondary metabolites (small-molecule extrolites). Some of these extrolites play a role in the infection process (50). Fumagillin and pseurotin A are also produced by A. fumigatus, and these two different types of extrolites are based on intertwined gene clusters (51) and may play a role in pathogenesis (50, 52, 53). While gliotoxin was not found in A. felis or A. udagawae, the latter species shared either helvolic acid, fumigatins, and fumitremorgins with A. fumigatus (52). There was a tendency for itraconazole-resistant strains to produce antafumicins and clavatols, at least within A. felis; however, two strains producing these extrolites were sensitive to itraconazole. Several studies have shown that the secondary metabolites produced by some filamentous fungi confer antifungal resistance (54). Here, we could find no correlation between the patterns of extrolites produced and the azole susceptibility of individual isolates. Additionally, none of the extrolites produced had a structure similar to that of the azoles. Nonetheless, the extrolites found could be further investigated, as previous studies looking at extrolite products in a limited number of isolates from the complex have demonstrated that they have unique properties, including antibacterial and anticancer activities (52, 55–58).

Other mechanisms of innate and acquired azole resistance should be further investigated as possible causes of AVSC resistance. Exploration of other resistance-associated genes in A. fumigatus, such as the *cyp51B* and genes involved in transcription, could help determine the cause of high azole MICs in AVSC isolates. The overexpression and induction of *cyp51B* were previously identified to be the most likely cause of azole resistance in two A. fumigatus isolates (30). Many other genes associated with reduced azole susceptibility to azole resistance in A. fumigatus could be explored in these species, including the gene for a truncated transcriptional regulator protein, *afyap1* (59, 60), and *aldA* (60), as could loss of the protein encoded by *alga* and a mutation in A. fumigatus *cox10* resulting in decreased azole absorption (61). Additionally, overexpression of the drug efflux mediator ATP-binding cassette transporter gene *atrF*, identified in azole-resistant A. fumigatus isolates (62–64), could be explored. Other genes that could be investigated include the *hapE* gene with the P88L mutation (*hapE* encodes a binding transcription factor complex subunit of the CCAAT binding complex [CBC]), shown to interfere with the binding of CBC to the target site (65), and the A. fumigatus multidrug resistance efflux pump genes *MDR3* and *MDR4*, whose overexpression has been associated with the induction of itraconazole resistance (63, 66). The sterol regulatory element binding protein SrbA, a transcriptional regulator in connection with the inactivation of the CBC, has been associated with the TR_34_ resistance mechanism in A. fumigatus by increasing sterol levels (65); however, it is less likely to be involved in the development of resistance in AVSC isolates, as TR_34_ was not present.

The amplification of *cyp51A* in members of the AVSC identified a number of nonsynonymous mutations within the group compared to the reference sequence for A. fumigatus; however, no mutations related to environmentally acquired resistance in A. fumigatus were identified. The single point mutation G138C, previously identified to confer multi- and pan-azole resistance in A. fumigatus clinical isolates associated with therapeutic exposure to azoles, was found in two environmental A. felis isolates (from woodland soil in Australia). *cyp51A* homology modeling of mutations found only in isolates with high azole MICs compared to the sequences of isolates with low azole MICs did not show a significant change in the overall protein structure or direct interference of the mutation with azole drug binding to confer resistance. Analysis of mutations in the *cyp51A* gene provides good phylogenetic information on the AVSC, but these mutations are unlikely to be a cause of azole resistance in isolates with high MICs of one or more azole drugs (95% of AVSC isolates tested). The mechanisms of innate resistance and other causes of acquired azole resistance should be further investigated for the AVSC. The growth of all AVSC isolates was inhibited by luliconazole and olorofim (F901318) on CLSI antifungal susceptibility testing, indicating that they show promise as potential therapeutic options.

## MATERIALS AND METHODS

### Isolates.

Fifty-six archived AVSC isolates were retrieved from the Fungal Culture Biobank (CBS) of the Westerdijk Fungal Biodiversity Institute, Utrecht, the Netherlands (*n* = 55), and from the National Mycology Reference Centre at SA Pathology, Adelaide, South Australia (*n* = 1). This included 6 human isolates, 20 animal isolates, 25 environmental isolates, and 5 isolates of unknown origin (see Table S1 in the supplemental material).

### Antifungal susceptibility testing.

Antifungal susceptibility testing was performed using the guidelines in the M38-A3 document of CLSI for the broth microdilution antifungal susceptibility method (*n* = 37) and MIC determination (67). Nineteen isolates failed to sporulate well enough to meet standardized conidial densities for inoculation; however, the *cyp51A* sequences were still determined for these isolates. Further, luliconazole (Nihon Nohyaku Co., Osaka, Japan) and olorofim (F2G, Manchester, UK) were dispensed into 96-well microtiter plates at a final concentration of 0.001 to 1 μg/ml. The final concentration of dimethyl sulfoxide in the test wells was ≤1%. As clinical breakpoints have not yet been assigned to AVSC species, interpretation of MICs did not include designation of isolates as susceptible or resistant. Instead, MICs were interpreted as high or low (17), informed by established epidemiological cutoff values (ECVs) for Aspergillus fumigatus (ECVs of itraconazole, voriconazole, and isavuconazole, 1 μg/ml; ECV of posaconazole, 0.5 μg/ml) (68, 69). Luliconazole and olorofim (F901318) MIC values were not assigned as susceptible or resistant, as neither clinical breakpoints nor ECVs have been established for these compounds against *Aspergillus* species, so instead, only MICs are reported.

Geometric mean, range, MIC_50_, and MIC_90_ values were calculated in Microsoft Office Excel (2013) software. Standard deviations and *t* test scores were calculated to determine if the triazole MICs for clinical isolates were higher than those for environmental isolates, with significance, determined using a two-tailed test, set at a *P *value of *<*0.05 (data not shown).

### *cyp51A* gene amplification and sequencing.

For all isolates, DNA extraction was performed using a MoBio DNA isolation kit (Sanbio B.V., Uden, the Netherlands). Multiple primer pairs were designed to amplify the *cyp51A* gene.

Primer design was based on 3 primer pairs previously used for amplification of the *cyp51A* gene in A. fumigatus by Chen et al. (2005): CYP 1-L, CYP 1-R, CYP 2-L, CYP 2-R, CYP 3-L, and CYP 3-R (84). From these, degenerate primers were designed using MEGA, version 6 (MEGA6), software (70). The *cyp51A* gene was located in the whole-genome sequence of AVSC member A. udagawae IFM 46973 (71), available in GenBank (National Center for Biotechnology Information, Bethesda, MD, USA [http://blast.ncbi.nlm.nih.gov/Blast.cgi]) (accession numbers BBXM01000001 to BBXM01001029 and BBXM01000040, region 670000-675000, contig Aud0040), and using MEGA6 software (70), a total of eight primer pairs were developed to amplify the *cyp51A* gene in AVSC members (Table 4). These were synthesized by Integrated DNA Technologies (Coralville, IA, USA).

**TABLE 4:** Primers used to target the *cyp51A* gene in AVSC members in this study

Primer sets for *cyp51A*	Primer pair	Sequence (5′ to 3′)
Aspergillus udagawae		
Forward 1	CYP0-F_uda^*a*^	GACTTTCATATCTTGCTCAGC
Reverse 1	CYP1-R_uda^*a*^	AGCCTTGAAAGTTCGGCGAG
Forward 2	CYP2-L^*b*^	CATGTGCCACTTATTGAGAAGG
Reverse 2	CYP2-R_uda^*a*^	CCTTGCGCATGAGCGAGTGA
Forward 3	CYP3-L _uda^*a*^	TTCCTCCGCTCCAGTACGAG
Reverse 3	CYP3-R_uda^*a*^	CCTTTGATGTCCTCGATGAAA
Forward 4	CYP3-L _uda^*a*^	TTCCTCCGCTCCAGTACGAG
Reverse 4	CYP4-R_uda^*a*^	GATCGCACCGTGTCCTTTG
Degenerate primers		
Forward 5	CYP0-F_deg^*c*^	GRCKTTCAWATSTTGCTCAGC
Reverse 5	CYP1-R_deg^*c*^	AGCCTTGAAARTTCGGYGAR
Forward 6	CYP2-L_deg^*c*^	CATGTGCCACTYATYGAGAAGG
Reverse 6	CYP2-R_deg^*c*^	CCTTGCGCATGAKMGAGTGA
Forward 7	CYP0-F_deg^*c*^	GRCKTTCAWATSTTGCTCAGC
Reverse 7	CYP2-R_deg^*c*^	CCTTGCGCATGAKMGAGTGA
Forward 8	CYP3-L^*b*^	TTCCTCCGCTCCAGTACAAG
Reverse 8	CYP4-R_deg^*c*^	GATCRCACCRWRTCCTTTG

a Primers identified from contig Aud0040 of A. udagawae (IFM 46973) (71) corresponding to the primers developed by Chen et al. (84).

b Primers used by Chen et al. (84).

c Degenerate primers developed for this study.

Amplification was performed using conventional PCR in a Life Technologies Applied Biosystems thermal cycler (model 2720; Life Technologies Europe BV, Bleiswijk, the Netherlands) for 5 min at 94°C; 35 cycles of 30 s at 94°C, 30 s at 52°C, and 1 min at 72°C; and then 5 min at 72°C and a hold at 10°C. In-house PCR product purification was performed in a Sensoquest lab cycler (Bioké, Leiden, the Netherlands), and Sanger sequencing was performed on a Hitachi/Applied Biosystems 3730xL genetic analyzer (Life Technologies [Thermo Fisher Scientific], USA).

The sequences were analyzed using the Seqman Pro tool in the DNASTAR program (version 12.1.0; Lasergene Molecular Biology and Lasergene Genomics, USA). Sequences were aligned in MEGA6 software using the MUSCLE alignment (70). Phylogenetic trees were constructed in MEGA6 software using the Kimura 2+ gamma distribution, the maximum likelihood discrete data method (tree searching method of 1,000 replicate trees), and bootstrapping (70).

Data were analyzed for mutations using the Fungal Resistance Database (FunResDB; The German National Reference Center for Invasive Fungal Infections, Jena, Germany), comparing the AVSC sequences with the sequence of the A. fumigatus reference strain with GenBank accession no. AF338659 (12). Mutations present in isolates with demonstrated azole resistance were compared to those present in other isolates of the same species, as well as those present in all isolates in the complex.

### *cyp51A* homology modeling.

The A. fumigatus *cyp51A* sequence (GenBank accession no. AF338659) was retrieved from Universal Protein Resources (http://www.uniprot.org), and a NCBI BLAST (blastp) search was performed to identify a suitable template. The crystal structure of Saccharomyces cerevisiae *cyp51A* (PDB accession number 5EQB) was selected as the template for homology modeling, as this was the *cyp51A* protein that returned the highest sequence identity in the blastp search (resolution, 2.19 Å; maximum and total score, 506; query cover, 94%; identity, 51%). Five homology models were produced based on the template using Schrödinger’s Prime program (72, 73), and the best model (model B) was selected after testing the quality of the models with the Verify 3D program (84.66% of the residues scored a three dimensional-one dimensional score of ≥0.2; Table S4) (74, 75). Homology models of A. felis (model F), A. viridinutans (model G), and A. aureolus (model H) were built on the basis of model B using the *cyp51A* amino acid sequences of isolates DTO 131-G1, DTO 050-F1, and DTO 278-B7, respectively (GenBank accession numbers are provided below, and the results obtained with the Verify 3D program are shown in Table S4). The models were verified with the Verify 3D program. Additionally, model F, the low-azole-MIC A. felis azole homology model, was subjected to mutations present in three A. felis isolates demonstrating high azole MICs (mutations T215S/G138C [model I] and S197C/Q340R [model J]; see Table 2 for isolate details). Finally, for models containing mutations at positions previously associated with azole resistance in A. fumigatus isolates (models H [V101] and I [G138]), mutations were added to A. fumigatus model B, producing model K (mutations from model I, G138C/T215S) and model L (mutations from model H, V101L/A103T/A234V/I360V/V428I/G505R/Q423D/F478V).

### Molecular docking and molecular mechanics-generalized Born surface area.

Models F to L were prepared for molecular docking using the Protein Preparation Wizard program (76). Two-dimensional (2D) structures of itraconazole, posaconazole, and voriconazole were first drawn using a 2D sketcher and then prepared with the LigPrep program (Schrödinger release 2017-3). Docking studies for the three triazoles with these models were carried out via the Glide program with standard precision (SP) (77–79) to determine the binding affinity between the ligand and the receptor. For each model, conformers of the triazoles that interact with the protein with the expected binding mode in the SP study were chosen to carry on to the next step. They were subjected to minimization via the MacroModel program (Schrödinger release 2017-3), followed by the ConfGen program (80), to generate alternative low-energy conformations. A docking study for these conformers was then carried out via the Glide program with extra precision (XP) (77–79). Only the conformers with the best docking score in the XP analysis were taken into consideration. The docking scores obtained from the Glide XP analysis of each azole-resistant receptor-ligand interaction were compared to those for their azole-susceptible counterpart. Since models G (based on Aspergillus viridinutans) and H (based on Aspergillus aureolus) do not have a corresponding low-azole-MIC model, they were also compared with model F. The results were interpreted to indicate that the more negative that the docking score value was, the better that the binding affinity was. The MMGBSA [Δ*G*(binding], ligand strain energy, and receptor strain energy of the ligand-receptor calculations were also determined using the Prime program (81), based on XP docking studies; residues within 20 Å from the ligand were set flexible. Default settings were used for all calculations. Δ*G*(binding) indicated how strong the binding was between the ligand and receptor, and the ligand and receptor strain energy indicated how much energy that the ligand and protein required to distort themselves into the appropriate binding pose.

### Root mean square deviation scores.

The differences between model F and azole-resistant models G to J were measured via superimposition and determination of RMSD scores for comparison of structural similarity.

### Extrolite profiling.

Extrolite extraction was performed on all isolates after growth on Czapek yeast agar and yeast extract sucrose agar at 25°C and 37°C for 7 days. Three agar plugs were extracted according to the agar plug extraction method of Smedsgaard (82). Extracts were analyzed using ultrahigh-performance liquid chromatography (UHPLC) with a diode array detector (Dionex Ultramate 3000 UHPLC), and the compounds were identified against an internal database of UV spectra and information in the literature (83). The extrolite standards reported by Nielsen et al. (83) were used.

### Data availability.

All sequences were submitted to GenBank and may be found under accession numbers MF178270 to MF178326 (https://www.ncbi.nlm.nih.gov/popset/?term=1388592591).

## Supplementary Material

Supplemental file 1

## References

[1] Ullmann AJ, Aguado JM, Arikan-Akdagli S, Denning DW, Groll AH, Lagrou K, Lass-Flörl C, Lewis RE, Munoz P, Verweij PE, Warris A, Ader F, Akova M, Arendrup MC, Barnes RA, Beigelman-Aubry C, Blot S, Bouza E, Brüggemann RJM, Buchheidt D, Cadranel J, Castagnola E, Chakrabarti A, Cuenca-Estrella M, Dimopoulos G, Fortun J, Gangneux J-P, Garbino J, Heinz WJ, Herbrecht R, Heussel CP, Kibbler CC, Klimko N, Kullberg BJ, Lange C, Lehrnbecher T, Löffler J, Lortholary O, Maertens J, Marchetti O, Meis JF, Pagano L, Ribaud P, Richardson M, Roilides E, Ruhnke M, Sanguinetti M, Sheppard DC, Sinkó J, Skiada A, Vehreschild MJGT, Viscoli C, Cornely OA 2018 Diagnosis and management of *Aspergillus* diseases: executive summary of the 2017 ESCMID-ECMM-ERS guideline. Clin Microbiol Infect 24(Suppl 1):e1–e38. doi:10.1016/j.cmi.2018.01.002. 29544767

[2] Verweij PE, Chowdhary A, Melchers WJG, Meis JF 2016 Azole resistance in Aspergillus fumigatus: can we retain the clinical use of mold-active antifungal azoles? Clin Infect Dis 62:362–368. doi:10.1093/cid/civ885. 26486705 PMC4706635

[3] Lestrade PP, Bentvelsen RG, Schauwvlieghe A, Schalekamp S, van der Velden W, Kuiper EJ, van Paassen J, van der Hoven B, van der Lee HA, Melchers WJG, de Haan AF, van der Hoeven HL, Rijnders BJA, van der Beek MT, Verweij PE 2018 Voriconazole resistance and mortality in invasive aspergillosis: a multicenter retrospective cohort study. Clin Infect Dis 68:1463–1471. doi:10.1093/cid/ciy859. 30307492

[4] Talbot JJ, Barrs VR 2018 One-health pathogens in the Aspergillus viridinutans complex. Med Mycol 56:1–12. doi:10.1093/mmy/myx016. 28379569

[5] van der Linden JW, Warris A, Verweij PE 2011 *Aspergillus* species intrinsically resistant to antifungal agents. Med Mycol 49:S82–S89. doi:10.3109/13693786.2010.499916. 20662634

[6] Denning DW, Venkateswarlu K, Oakley KL, Anderson MJ, Manning NJ, Stevens DA, Warnock DW, Kelly SL 1997 Itraconazole resistance in *Aspergillus fumigatus*. Antimicrob Agents Chemother 41:1364–1368. doi:10.1128/AAC.41.6.1364. 9174200 PMC163916

[7] Chowdhary A, Kathuria S, Xu J, Meis JF 2013 Emergence of azole-resistant *Aspergillus fumigatus* strains due to agricultural azole use creates an increasing threat to human health. PLoS Pathog 9:e1003633. doi:10.1371/journal.ppat.1003633. 24204249 PMC3812019

[8] Snelders E, van der Lee HA, Kuijpers J, Rijs AJ, Varga J, Samson RA, Mellado E, Donders AR, Melchers WJ, Verweij PE 2008 Emergence of azole resistance in *Aspergillus fumigatus* and spread of a single resistance mechanism. PLoS Med 5:e219. doi:10.1371/journal.pmed.0050219. 18998768 PMC2581623

[9] Snelders E, Camps SM, Karawajczyk A, Schaftenaar G, Kema GH, van der Lee HA, Klaassen CH, Melchers WJ, Verweij PE 2012 Triazole fungicides can induce cross-resistance to medical triazoles in *Aspergillus fumigatus*. PLoS One 7:e31801. doi:10.1371/journal.pone.0031801. 22396740 PMC3291550

[10] Chowdhary A, Sharma C, Meis JF 2017 Azole-resistant aspergillosis: epidemiology, molecular mechanisms, and treatment. J Infect Dis 216:S436–S444. doi:10.1093/infdis/jix210. 28911045

[11] Chowdhary A, Meis JF 2018 Emergence of azole resistant *Aspergillus fumigatus* and One Health: time to implement environmental stewardship. Environ Microbiol 20:1299–1301. doi:10.1111/1462-2920.14055. 29393565

[12] Weber M, Schaer J, Walther G, Kaerger K, Steinmann J, Rath P, Spiess B, Buchheidt D, Hamprecht A, Kurzai O 2018 FunResDB—a web resource for genotypic susceptibility testing of *Aspergillus fumigatus*. Med Mycol 56:117–120. doi:10.1093/mmy/myx015. 28340175 PMC5896429

[13] van der Linden JW, Camps SM, Kampinga GA, Arends JP, Debets-Ossenkopp YJ, Haas PJ, Rijnders BJ, Kuijper EJ, van Tiel FH, Varga J, Karawajczyk A, Zoll J, Melchers WJ, Verweij PE 2013 Aspergillosis due to voriconazole highly resistant *Aspergillus fumigatus* and recovery of genetically related resistant isolates from domiciles. Clin Infect Dis 57:513–520. doi:10.1093/cid/cit320. 23667263

[14] Howard SJ, Arendrup MC 2011 Acquired antifungal drug resistance in *Aspergillus fumigatus*: epidemiology and detection. Med Mycol 49:S90–S95. doi:10.3109/13693786.2010.508469. 20795765

[15] Hubka V, Barrs V, Dudová Z, Sklenář F, Kubátová A, Matsuzawa T, Yaguchi T, Horie Y, Nováková A, Frisvad JC, Talbot JJ, Kolařík M 2018 Unravelling species boundaries in the *Aspergillus viridinutans* complex (section *Fumigati*): opportunistic human and animal pathogens capable of interspecific hybridization. Persoonia 41:142–174. doi:10.3767/persoonia.2018.41.08. 30728603 PMC6344812

[16] Talbot JJ, Houbraken J, Frisvad C, Samson RA, Kidd SE, Pitt J, Lindsay S, Beatty JA, Barrs VR 2017 Discovery of *Aspergillus frankstonensis* sp. nov. during environmental sampling for animal and human fungal pathogens. PLoS One 12:e0181660. doi:10.1371/journal.pone.0181660. 28792943 PMC5549889

[17] Lyskova P, Hubka V, Svobodova L, Barrs V, Dhand NK, Yaguchi T, Matsuzawa T, Horie Y, Kolarik M, Dobias R, Hamal P 2018 Antifungal susceptibility of the *Aspergillus viridinutans* complex: comparison of two *in vitro* methods. Antimicrob Agents Chemother 62:e01927-17. doi:10.1128/AAC.01927-17. 29437620 PMC5913995

[18] Zargaran M, Taghipour S, Kiasat N, Aboualigalehdari E, Rezaei-Matehkolaei A, Zarei Mahmoudabadi A, Shamsizadeh F 2017 Luliconazole, an alternative antifungal agent against *Aspergillus terreus*. J Mycol Med 27:351–356. doi:10.1016/j.mycmed.2017.04.011. 28483449

[19] Abastabar M, Rahimi N, Meis JF, Aslani N, Khodavaisy S, Nabili M, Rezaei-Matehkolaei A, Makimura K, Badali H 2016 Potent activities of novel imidazoles lanoconazole and luliconazole against a collection of azole-resistant and -susceptible *Aspergillus fumigatus* strains. Antimicrob Agents Chemother 60:6916–6919. doi:10.1128/AAC.01193-16. 27572389 PMC5075107

[20] Niwano Y, Kuzuhara N, Goto Y, Munechika Y, Kodama H, Kanai K, Yoshida M, Miyazaki T, Yamaguchi H 1999 Efficacy of NND-502, a novel imidazole antimycotic agent, in experimental models of *Candida albicans* and *Aspergillus fumigatus* infections. Int J Antimicrob Agents 12:221–228. doi:10.1016/S0924-8579(99)00076-X. 10461840

[21] Biswas C, Law D, Birch M, Halliday C, Sorrell TC, Rex J, Slavin M, Chen SC 2018 In vitro activity of the novel antifungal compound F901318 against Australian Scedosporium and Lomentospora fungi. Med Mycol 56:1050–1054. doi:10.1093/mmy/myx161. 29370408

[22] Buil JB, Rijs A, Meis JF, Birch M, Law D, Melchers WJG, Verweij PE 2017 In vitro activity of the novel antifungal compound F901318 against difficult-to-treat *Aspergillus* isolates. J Antimicrob Chemother 72:2548–2552. doi:10.1093/jac/dkx177. 28605488

[23] Hope WW, McEntee L, Livermore J, Whalley S, Johnson A, Farrington N, Kolamunnage-Dona R, Schwartz J, Kennedy A, Law D, Birch M, Rex JH 2017 Pharmacodynamics of the orotomides against Aspergillus fumigatus: new opportunities for treatment of multidrug-resistant fungal disease. mBio 8:e01157-17. doi:10.1128/mBio.01157-17. 28830945 PMC5565967

[24] Negri CE, Johnson A, McEntee L, Box H, Whalley S, Schwartz JA, Ramos-Martin V, Livermore J, Kolamunnage-Dona R, Colombo AL, Hope WW 2018 Pharmacodynamics of the novel antifungal agent F901318 for acute sinopulmonary aspergillosis caused by *Aspergillus flavus*. J Infect Dis 217:1118–1127. doi:10.1093/infdis/jix479. 28968675 PMC5909626

[25] Oliver JD, Sibley GE, Beckmann N, Dobb KS, Slater MJ, McEntee L, Du Pre S, Livermore J, Bromley MJ, Wiederhold NP, Hope WW, Kennedy AJ, Law D, Birch M 2016 F901318 represents a novel class of antifungal drug that inhibits dihydroorotate dehydrogenase. Proc Natl Acad Sci U S A 113:12809–12814. doi:10.1073/pnas.1608304113. 27791100 PMC5111691

[26] Kidd SE, Goeman E, Meis JF, Slavin MA, Verweij PE 2015 Multi-triazole-resistant *Aspergillus fumigatus* infections in Australia. Mycoses 58:350–355. doi:10.1111/myc.12324. 25885568

[27] Snelders E, Karawajczyk A, Schaftenaar G, Verweij PE, Melchers WJ 2010 Azole resistance profile of amino acid changes in *Aspergillus fumigatus CYP51A* based on protein homology modeling. Antimicrob Agents Chemother 54:2425–2430. doi:10.1128/AAC.01599-09. 20385860 PMC2876375

[28] Abdolrasouli A, Rhodes J, Beale MA, Hagen F, Rogers TR, Chowdhary A, Meis JF, Armstrong-James D, Fisher MC 2015 Genomic context of azole resistance mutations in *Aspergillus fumigatus* determined using whole-genome sequencing. mBio 6:e00536-15. doi:10.1128/mBio.00536-15. 26037120 PMC4453006

[29] Albarrag AM, Anderson MJ, Howard SJ, Robson GD, Warn PA, Sanglard D, Denning DW 2011 Interrogation of related clinical pan-azole-resistant *Aspergillus fumigatus* strains: G138C, Y431C, and G434C single nucleotide polymorphisms in *cyp51A*, upregulation of *cyp51A*, and integration and activation of transposon *Atf1* in the *cyp51A* promoter. Antimicrob Agents Chemother 55:5113–5121. doi:10.1128/AAC.00517-11. 21876055 PMC3195044

[30] Buied A, Moore CB, Denning DW, Bowyer P 2013 High-level expression of cyp51B in azole-resistant clinical *Aspergillus fumigatus* isolates. J Antimicrob Chemother 68:512–514. doi:10.1093/jac/dks451. 23208831

[31] Escribano P, Recio S, Pelaez T, Bouza E, Guinea J 2011 *Aspergillus fumigatus* strains with mutations in the *cyp51A* gene do not always show phenotypic resistance to itraconazole, voriconazole, or posaconazole. Antimicrob Agents Chemother 55:2460–2462. doi:10.1128/AAC.01358-10. 21321141 PMC3088257

[32] Garcia-Effron G, Dilger A, Alcazar-Fuoli L, Park S, Mellado E, Perlin DS 2008 Rapid detection of triazole antifungal resistance in *Aspergillus fumigatus*. J Clin Microbiol 46:1200–1206. doi:10.1128/JCM.02330-07. 18234874 PMC2292958

[33] Howard SJ, Cerar D, Anderson MJ, Albarrag A, Fisher MC, Pasqualotto AC, Laverdiere M, Arendrup MC, Perlin DS, Denning DW 2009 Frequency and evolution of azole resistance in *Aspergillus fumigatus* associated with treatment failure. Emerg Infect Dis 15:1068–1076. doi:10.3201/eid1507.090043. 19624922 PMC2744247

[34] Howard SJ, Webster I, Moore CB, Gardiner RE, Park S, Perlin DS, Denning DW 2006 Multi-azole resistance in *Aspergillus fumigatus*. Int J Antimicrob Agents 28:450–453. doi:10.1016/j.ijantimicag.2006.08.017. 17034993

[35] Lavergne RA, Morio F, Favennec L, Dominique S, Meis JF, Gargala G, Verweij PE, Le Pape P 2015 First description of azole-resistant *Aspergillus fumigatus* due to TR46/Y121F/T289A mutation in France. Antimicrob Agents Chemother 59:4331–4335. doi:10.1128/AAC.00127-15. 25918139 PMC4468656

[36] Mavridou E, Meletiadis J, Rijs A, Mouton JW, Verweij PE 2015 The strength of synergistic interaction between posaconazole and caspofungin depends on the underlying azole resistance mechanism of *Aspergillus fumigatus*. Antimicrob Agents Chemother 59:1738–1744. doi:10.1128/AAC.04469-14. 25583716 PMC4325813

[37] Meneau I, Coste AT, Sanglard D 2016 Identification of *Aspergillus fumigatus* multidrug transporter genes and their potential involvement in antifungal resistance. Med Mycol 54:616–627. doi:10.1093/mmy/myw005. 26933209

[38] Mortensen KL, Jensen RH, Johansen HK, Skov M, Pressler T, Howard SJ, Leatherbarrow H, Mellado E, Arendrup MC 2011 *Aspergillus* species and other molds in respiratory samples from patients with cystic fibrosis: a laboratory-based study with focus on *Aspergillus fumigatus* azole resistance. J Clin Microbiol 49:2243–2251. doi:10.1128/JCM.00213-11. 21508152 PMC3122734

[39] van Ingen J, van der Lee HA, Rijs AJ, Snelders E, Melchers WJ, Verweij PE 2015 High-level pan-azole-resistant aspergillosis. J Clin Microbiol 53:2343–2345. doi:10.1128/JCM.00502-15. 25903576 PMC4473200

[40] Wiederhold NP, Gil VG, Gutierrez F, Lindner JR, Albataineh MT, McCarthy DI, Sanders C, Fan H, Fothergill AW, Sutton DA 2016 First detection of TR34 L98H and TR46 Y121F T289A *cyp51* mutations in *Aspergillus fumigatus* isolates in the United States. J Clin Microbiol 54:168–171. doi:10.1128/JCM.02478-15. 26491179 PMC4702720

[41] Garcia-Rubio R, Alcazar-Fuoli L, Monteiro MC, Monzon S, Cuesta I, Pelaez T, Mellado E 2018 Insight into the significance of *Aspergillus fumigatus cyp51A* polymorphisms. Antimicrob Agents Chemother 62:e00241-18. doi:10.1128/AAC.00241-18. 29632011 PMC5971592

[42] Mellado E, Alcazar-Fuoli L, Cuenca-Estrella M, Rodriguez-Tudela JL 2011 Role of *Aspergillus lentulus* 14-alpha sterol demethylase (*cyp51A*) in azole drug susceptibility. Antimicrob Agents Chemother 55:5459–5468. doi:10.1128/AAC.05178-11. 21947395 PMC3232793

[43] Mellado E, Garcia-Effron G, Buitrago MJ, Alcazar-Fuoli L, Cuenca-Estrella M, Rodriguez-Tudela JL 2005 Targeted gene disruption of the 14-alpha sterol demethylase (cyp51A) in Aspergillus fumigatus and its role in azole drug susceptibility. Antimicrob Agents Chemother 49:2536–2538. doi:10.1128/AAC.49.6.2536-2538.2005. 15917566 PMC1140498

[44] Umeyama T, Hayashi Y, Shimosaka H, Inukai T, Yamagoe S, Takatsuka S, Hoshino Y, Nagi M, Nakamura S, Kamei K, Ogawa K, Miyazaki Y 2018 CRISPR/Cas9 genome editing to demonstrate the contribution of Cyp51A Gly138Ser to azole resistance in Aspergillus fumigatus. Antimicrob Agents Chemother 62:e00894-18. doi:10.1128/AAC.00894-18. 29914956 PMC6125507

[45] Al Abdallah Q, Ge W, Fortwendel JR 2017 A simple and universal system for gene manipulation in Aspergillus fumigatus: in vitro-assembled Cas9-guide RNA ribonucleoproteins coupled with microhomology repair templates. mSphere 2:e00446-17. doi:10.1128/mSphere.00446-17. 29202040 PMC5700375

[46] Sharma C, Hagen F, Moroti R, Meis JF, Chowdhary A 2015 Triazole-resistant *Aspergillus fumigatus* harbouring G54 mutation: is it de novo or environmentally acquired? J Glob Antimicrob Resist 3:69–74. doi:10.1016/j.jgar.2015.01.005. 27873672

[47] Bader O, Tünnermann J, Dudakova A, Tangwattanachuleeporn M, Weig M, Groß U 2015 Environmental isolates of azole-resistant *Aspergillus fumigatus* in Germany. Antimicrob Agents Chemother 59:4356–4359. doi:10.1128/AAC.00100-15. 25941229 PMC4468681

[48] Liu M, Zheng N, Li D, Zheng H, Zhang L, Ge H, Liu W 2016 cyp51A-based mechanism of azole resistance in *Aspergillus fumigatus*: illustration by a new 3D structural model of *Aspergillus fumigatus CYP51A* protein. Med Mycol 54:400–408. doi:10.1093/mmy/myv102. 26768370

[49] Mortensen KL, Mellado E, Lass-Flörl C, Rodriguez-Tudela JL, Johansen HK, Arendrup MC 2010 Environmental study of azole-resistant *Aspergillus fumigatus* and other aspergilli in Austria, Denmark, and Spain. Antimicrob Agents Chemother 54:4545–4549. doi:10.1128/AAC.00692-10. 20805399 PMC2976122

[50] Keller NP 2015 Translating biosynthetic gene clusters into fungal armor and weaponry. Nat Chem Biol 11:671–677. doi:10.1038/nchembio.1897. 26284674 PMC4682562

[51] Wiemann P, Guo CJ, Palmer JM, Sekonyela R, Wang CC, Keller NP 2013 Prototype of an intertwined secondary-metabolite supercluster. Proc Natl Acad Sci U S A 110:17065–17070. doi:10.1073/pnas.1313258110. 24082142 PMC3801025

[52] Frisvad JC, Larsen TO 2016 Extrolites of *Aspergillus fumigatus* and other pathogenic species in *Aspergillus* section *Fumigati*. Front Microbiol 6:1485. doi:10.3389/fmicb.2015.01485. 26779142 PMC4703822

[53] Lind AL, Wisecaver JH, Lameiras C, Wiemann P, Palmer JM, Keller NP, Rodrigues F, Goldman GH, Rokas A 2017 Drivers of genetic diversity in secondary metabolic gene clusters within a fungal species. PLoS Biol 15:e2003583. doi:10.1371/journal.pbio.2003583. 29149178 PMC5711037

[54] Keller NP 2019 Fungal secondary metabolism: regulation, function and drug discovery. Nat Rev Microbiol 17:167–180. doi:10.1038/s41579-018-0121-1. 30531948 PMC6381595

[55] Lillehoj EB, Ciegler A 1972 A toxic substance from *Aspergillus viridi-nutans*. Can J Microbiol 18:193–197. doi:10.1139/m72-030. 4622987

[56] Lillehoj EB, Milburn MS 1973 Viriditoxin production by *Aspergillus viridi-nutans* and related species. Appl Microbiol 26:202–205. 4582816 10.1128/am.26.2.202-205.1973 PMC379751

[57] Mendes G, Gonçalves VN, Souza-Fagundes EM, Kohlhoff M, Rosa CA, Zani CL, Cota BB, Rosa LH, Johann S 2016 Antifungal activity of extracts from Atacama Desert fungi against *Paracoccidioides brasiliensis* and identification of *Aspergillus felis* as a promising source of natural bioactive compounds. Mem Inst Oswaldo Cruz 111:209–217. doi:10.1590/0074-02760150451. 27008375 PMC4804504

[58] Gonçalves VN, Cantrell CL, Wedge DE, Ferreira MC, Soares MA, Jacob MR, Oliveira FS, Galante D, Rodrigues F, Alves TM, Zani CL, Junior PA, Murta S, Romanha AJ, Barbosa EC, Kroon EG, Oliveira JG, Gomez-Silva B, Galetovic A, Rosa CA, Rosa LH 2016 Fungi associated with rocks of the Atacama Desert: taxonomy, distribution, diversity, ecology and bioprospection for bioactive compounds. Environ Microbiol 18:232–245. doi:10.1111/1462-2920.13005. 26235221

[59] Qiao J, Liu W, Li R 2010 Truncated Afyap1 attenuates antifungal susceptibility of Aspergillus fumigatus to voriconazole and confers adaptation of the fungus to oxidative stress. Mycopathologia 170:155–160. doi:10.1007/s11046-010-9309-2. 20376564

[60] Hagiwara D, Takahashi H, Watanabe A, Takahashi-Nakaguchi A, Kawamoto S, Kamei K, Gonoi T 2014 Whole-genome comparison of Aspergillus fumigatus strains serially isolated from patients with aspergillosis. J Clin Microbiol 52:4202–4209. doi:10.1128/JCM.01105-14. 25232160 PMC4313286

[61] Wei X, Chen P, Gao R, Li Y, Zhang A, Liu F, Lu L 2016 Screening and characterization of a non-cyp51A mutation in an Aspergillus fumigatus cox10 strain conferring azole resistance. Antimicrob Agents Chemother 61:e02101-16. doi:10.1128/AAC.02101-16. 27799210 PMC5192120

[62] Slaven JW, Anderson MJ, Sanglard D, Dixon GK, Bille J, Roberts IS, Denning DW 2002 Increased expression of a novel Aspergillus fumigatus ABC transporter gene, atrF, in the presence of itraconazole in an itraconazole resistant clinical isolate. Fungal Genet Biol 36:199–206. doi:10.1016/S1087-1845(02)00016-6. 12135575

[63] da Silva Ferreira ME, Capellaro JL, dos Reis Marques E, Malavazi I, Perlin D, Park S, Anderson JB, Colombo AL, Arthington-Skaggs BA, Goldman MHS, Goldman GH 2004 In vitro evolution of itraconazole resistance in Aspergillus fumigatus involves multiple mechanisms of resistance. Antimicrob Agents Chemother 48:4405–4413. doi:10.1128/AAC.48.11.4405-4413.2004. 15504870 PMC525395

[64] Bowyer P, Mosquera J, Anderson M, Birch M, Bromley M, Denning DW 2012 Identification of novel genes conferring altered azole susceptibility in Aspergillus fumigatus. FEMS Microbiol Lett 332:10–19. doi:10.1111/j.1574-6968.2012.02575.x. 22509997 PMC4220099

[65] Gsaller F, Hortschansky P, Furukawa T, Carr PD, Rash B, Capilla J, Muller C, Bracher F, Bowyer P, Haas H, Brakhage AA, Bromley MJ 2016 Sterol biosynthesis and azole tolerance is governed by the opposing actions of SrbA and the CCAAT binding complex. PLoS Pathog 12:e1005775. doi:10.1371/journal.ppat.1005775. 27438727 PMC4954732

[66] Nascimento AM, Goldman GH, Park S, Marras SA, Delmas G, Oza U, Lolans K, Dudley MN, Mann PA, Perlin DS 2003 Multiple resistance mechanisms among *Aspergillus fumigatus* mutants with high-level resistance to itraconazole. Antimicrob Agents Chemother 47:1719–1726. doi:10.1128/aac.47.5.1719-1726.2003. 12709346 PMC153329

[67] Clinical and Laboratory Standards Institute. 2017 Reference method for broth dilution antifungal susceptibility testing of filamentous fungi, 3rd ed. M38-A3 Clinical and Laboratory Standards Institute, Wayne, PA.

[68] Espinel-Ingroff A, Diekema DJ, Fothergill A, Johnson E, Pelaez T, Pfaller MA, Rinaldi MG, Canton E, Turnidge J 2010 Wild-type MIC distributions and epidemiological cutoff values for the triazoles and six *Aspergillus* spp. for the CLSI broth microdilution method (M38-A2 document). J Clin Microbiol 48:3251–3257. doi:10.1128/JCM.00536-10. 20592159 PMC2937688

[69] Clinical and Laboratory Standards Institute. 2018 Epidemiology cutoff values of antifungal susceptibility testing, 2nd ed. CLSI supplement M59 Clinical and Laboratory Standards Institute, Wayne, PA.

[70] Tamura K, Stecher G, Peterson D, Filipski A, Kumar S 2013 MEGA6: molecular evolutionary genetics analysis version 6.0. Mol Biol Evol 30:2725–2729. doi:10.1093/molbev/mst197. 24132122 PMC3840312

[71] Kusuya Y, Takahashi-Nakaguchi A, Takahashi H, Yaguchi T 2015 Draft genome sequence of the pathogenic filamentous fungus *Aspergillus udagawae* strain IFM 46973^T^. Genome Announc 3:e00834-15. doi:10.1128/genomeA.00834-15.. 26251487 PMC4541281

[72] Jacobson MP, Pincus DL, Rapp CS, Day TJF, Honig B, Shaw DE, Friesner RA 2004 A hierarchical approach to all-atom protein loop prediction. Proteins 55:351–367. doi:10.1002/prot.10613. 15048827

[73] Jacobson MP, Friesner RA, Xiang Z, Honig B 2002 On the role of crystal packing forces in determining protein sidechain conformations. J Mol Biol 320:597–608. doi:10.1016/S0022-2836(02)00470-9. 12096912

[74] Bowie JU, Luthy R, Eisenberg D 1991 A method to identify protein sequences that fold into a known three-dimensional structure. Science 253:164–170. doi:10.1126/science.1853201. 1853201

[75] Luthy R, Bowie JU, Eisenberg D 1992 Assessment of protein models with three-dimensional profiles. Nature 356:83–85. doi:10.1038/356083a0. 1538787

[76] Sastry GM, Adzhigirey M, Day T, Annabhimoju R, Sherman W 2013 Protein and ligand preparation: parameters, protocols, and influence on virtual screening enrichments. J Comput Aided Mol Des 27:221–234. doi:10.1007/s10822-013-9644-8. 23579614

[77] Friesner RA, Murphy RB, Repasky MP, Frye LL, Greenwood JR, Halgren TA, Sanschagrin PC, Mainz DT 2006 Extra precision Glide: docking and scoring incorporating a model of hydrophobic enclosure for protein-ligand complexes. J Med Chem 49:6177–6196. doi:10.1021/jm051256o. 17034125

[78] Halgren TA, Murphy RB, Friesner RA, Beard HS, Frye LL, Pollard WT, Banks JL 2004 Glide: a new approach for rapid, accurate docking and scoring. 2. Enrichment factors in database screening. J Med Chem 47:1750–1759. doi:10.1021/jm030644s. 15027866

[79] Friesner RA, Banks JL, Murphy RB, Halgren TA, Klicic JJ, Mainz DT, Repasky MP, Knoll EH, Shaw DE, Shelley M, Perry JK, Francis P, Shenkin PS 2004 Glide: a new approach for rapid, accurate docking and scoring. 1. Method and assessment of docking accuracy. J Med Chem 47:1739–1749. doi:10.1021/jm0306430. 15027865

[80] Watts KS, Dalal P, Murphy RB, Sherman W, Friesner RA, Shelley JC 2010 ConfGen: a conformational search method for efficient generation of bioactive conformers. J Chem Inf Model 50:534–546. doi:10.1021/ci100015j. 20373803

[81] Li J, Abel R, Zhu K, Cao Y, Zhao S, Friesner RA 2011 The VSGB 2.0 model: a next generation energy model for high resolution protein structure modeling. Proteins 79:2794–2812. doi:10.1002/prot.23106. 21905107 PMC3206729

[82] Smedsgaard J 1997 Micro-scale extraction procedure for standardized screening of fungal metabolite production in cultures. J Chromatogr A 760:264–270. doi:10.1016/s0021-9673(96)00803-5. 9062989

[83] Nielsen ML, Nielsen JB, Rank C, Klejnstrup ML, Holm DK, Brogaard KH, Hansen BG, Frisvad JC, Larsen TO, Mortensen UH 2011 A genome-wide polyketide synthase deletion library uncovers novel genetic links to polyketides and meroterpenoids in *Aspergillus nidulans*. FEMS Microbiol Lett 321:157–166. doi:10.1111/j.1574-6968.2011.02327.x. 21658102

[84] Chen J, Li H, Li R, Bu D, Wan Z 2005 Mutations in the cyp51A gene and susceptibility to itraconazole in *Aspergillus fumigatus* serially isolated from a patient with lung aspergilloma. J Antimicrob Chemother 55:31–37. doi:10.1093/jac/dkh507. 15563516

