# Supplementary material for: cyp51A Mutations, Extrolite Profiles, and Antifungal Susceptibility in Clinical and Environmental Isolates of the Aspergillus viridinutans Species Complex
Source: Antimicrob Agents Chemother. 2019 Oct 22;63(11):e00632-19. doi: 10.1128/AAC.00632-19 (PMC6811395; doi:10.1128/AAC.00632-19)
Supplement: Supplemental file 1 [file AAC.00632-19-s0001.pdf]

Supplementary Tables

Supplementary Table 1. All isolates used in this study.

**Strain number**	**Species**	**Source**	**Location**	**Antifungal susceptibility testing**
DTO 019-F2	*A. arcoverdensis*	soil	Australia	-
DTO 316-F7 (= CBS 139187 (T))	*A. arcoverdensis*	semi-desert soil	Brazil	-
DTO 316-F9 (= CBS 139188)	*A. arcoverdensis*	unknown	Brazil	CLSI
DTO 278-B7 (= CBS 137453)	*A. aureolus*	soil	Brazil	CLSI
DTO 331-G6 (= CBS 105.55 (T) = DTO 052-C8 = NRRL 2244)	*A. aureolus*	soil	Ghana	-
CBS 143249	*A. felis*	human	Australia	CLSI
DTO 131-E3	*A. felis*	cat, RBM	Australia	-
DTO 131-E4	*A. felis*	cat, RBM	Australia	CLSI
DTO 131-E5	*A. felis*	cat, RBM	Australia	CLSI
DTO 131-E6 (= CBS 130244)	*A. felis*	cat, RBM	Australia	CLSI
DTO 131-E9	*A. felis*	cat, RBM	Australia	-
DTO 131-F1	*A. felis*	cat, RBM	Australia	CLSI
DTO 131-F2	*A. felis*	cat, RBM	Australia	-
DTO 131-F3	*A. felis*	cat, RBM	Australia	CLSI
DTO 131-F4 (= CBS 130245 (T))	*A. felis*	cat, RBM	Australia	-
DTO 131-F6	*A. felis*	cat, RBM	Australia	CLSI
DTO 131-F9 (= CBS 130246)	*A. felis*	cat, SNC	Australia	-
DTO 131-G1	*A. felis*	cat, RBM	Australia	CLSI
DTO 131-G2 (= CBS 130247)	*A. felis*	cat, RBM	Australia	-
DTO 131-G3 (= CBS 130248)	*A. felis*	cat, RBM	Australia	CLSI
DTO 155-G3 (= CBS 130249)	*A. felis*	dog, VH	Australia	CLSI
DTO 159-C9 (= CBS 130250)	*A. felis*	cat, RBM	United Kingdom	-

DTO 175-H3	*A. felis*	surface water	Portugal	CLSI
DTO 176-F1	*A. felis*	air	Germany	CLSI
DTO 316-C8	*A. felis*	CBS culture contaminant	The Netherlands	CLSI
DTO 327-G4	*A. felis*	human patient	The Netherlands	CLSI
DTO 341-E4	*A. felis*	woodland soil	Frankston, Australia	CLSI
DTO 341-E5 (= CBS 142232)	*A. felis*	woodland soil	Frankston, Australia	CLSI
DTO 341-E6	*A. felis*	woodland soil	Frankston, Australia	-
DTO 341-E8	*A. felis*	woodland soil	Frankston, Australia	CLSI
DTO 341-E9	*A. felis*	woodland soil	Frankston, Australia	CLSI
DTO 341-F1	*A. felis*	woodland soil	Frankston, Australia	-
DTO 341-F2	*A. felis*	woodland soil	Frankston, Australia	CLSI
DTO 342-I4 (= NRRL 62900 = CM-3147 = CBS 140762 (T))	*A. felis*	human, OPE	Spain	CLSI
DTO 342-I5 (= NRRL 62901 = CM-5623 = CBS 140765)	*A. felis*	human, lungs	Portugal	CLSI
DTO 342-I6 (= NRRL 62902 = CM-4518 = CBS 140766)	*A. felis*	human, nail	Spain	CLSI
DTO 342-I7 (= NRRL 62903 = CM-6087 = CBS 140763 (T))	*A. felis*	human, sputum	Spain	CLSI
DTO 341-E7 (= CBS 142233 = IBT 34172 (T))	*A. frankstonensis*	woodland soil	Frankston, Australia	-
DTO 341-F3 (= CBS 142234 = IBT 34204)	*A. frankstonensis*	woodland soil	Frankston, Australia	CLSI
DTO 153-A1 (= CBS 458.75)	*A. pseudoviridinutans*	soil	India	-
DTO 303-A1	*A. pseudoviridinutans*	*Pinus caribea* (pine tree)	Sri Lanka	CLSI
DTO 304-I5 (= NRRL 62904 = NIHAV1 = CBS 140396 (T))	*A. pseudoviridinutans*	human, lung	USA	-
DTO 342-I3 (= NRRL 6106 = CBS 140764)	*A. pseudoviridinutans*	unknown	unknown	CLSI
DTO 278-B6 (= CBS 137452 (T))	*A. siamensis*	soil	Thailand	-
DTO 006-A3	*A. udagawae*	soil	USA	CLSI
DTO 019-D7	*A. udagawae*	unknown	unknown	-
DTO 019-D8	*A. udagawae*	unknown	unknown	CLSI
DTO 157-D7 (= CBS 114217 (T))	*A. udagawae*	soil	Brazil	CLSI
DTO 157-D8 (= CBS 114218)	*A. udagawae*	soil	Brazil	CLSI

DTO 166-D6	*A. udagawae*	cat	Australia	CLSI
DTO 283-D3	*A. udagawae*	soil	Thailand	-
DTO 308-H6	*A. udagawae*	soil	Turkey	CLSI
DTO 341-E3 (= CBS 142231)	*A. udagawae*	cat, RBM	Kealba, Australia	CLSI
DTO 050-F1 (= CBS 127.56 (T) = NRRL 4365)	*A. viridinutans*	rabbit dung	Australia	CLSI
DTO 155-G2	*A. wyomingensis*	cat, RBM	Australia	-
DTO 332-B1 (= CBS 135456 (T))	*A. wyomingensis*	coal mine reclamation site soil	Glenrock, USA	CLSI

Abbreviations: (T), type strain; CLSI, Clinical Laboratory Standards Institute; - = isolate did not sporulate for testing.

Supplementary Table 2. CLSI antifungal susceptibility testing results for 37 *Aspergillus viridinutans* species complex isolates included in this study.

**Drug**	**Species**	**MIC/MEC (µg/mL) distribution among tested isolates**																
		**0.001**	**0.002**	**0.004**	**0.008**	**0.015**	**0.03**	**0.06**	**0.125**	**0.25**	**0.5**	**1**	**2**	**4**	**8**	**16**	**>16**	**GM**
**ITZ***	*A. arcoverdensis*									1								
	A. aureolus									1								
	A. felis								1	2	4	1				1	14	4.38
	A. frankstonensis																1	
	A. pseudoviridinutans									1							1	
	A. udagawae									1	5						1	
	A. viridinutans																1	
	A. wyomingensis																1	
**VCZ***	*A. arcoverdensis*										1							
	A. aureolus										1							
	A. felis									1	2		4	13	3			2.87
	A. frankstonensis														1			

	A. pseudoviridinutans										1			1				
	A. udagawae												5	1		1		
	A. viridinutans													1				
	A. wyomingensis													1				
**POS***	*A. arcoverdensis*							1										
	A. aureolus										1							
	A. felis						2		4	15	2							0.2
	A. frankstonensis										1							
	A. pseudoviridinutans							1		1								
	A. udagawae								3	3							1	
	A. viridinutans									1								
	A. wyomingensis									1								
**ISA***	*A. arcoverdensis*											1						
	A. aureolus								1									
	A. felis										2	1	6	12	2			2.79
	A. frankstonensis														1			
	A. pseudoviridinutans										1			1				
	A. udagawae											1	5		1			
	A. viridinutans													1				
	A. wyomingensis														1			
**AMB***	*A. arcoverdensis*								1									
	A. aureolus											1						
	A. felis									2	1	14	6					1.03
	A. frankstonensis												1					
	A. pseudoviridinutans								1			1						
	A. udagawae										1	3	3					
	A. viridinutans										1							
	A. wyomingensis											1						

**MIF†**	*A. arcoverdensis*								1									
	A. aureolus											1						
	A. felis						4	5	3	2	1	1			1		6	0.44
	A. frankstonensis							1										
	A. pseudoviridinutans							2										
	A. udagawae							3	1	1	1		1					
	A. viridinutans								1									
	A. wyomingensis																1	
**LUL***	*A. arcoverdensis*	1																
	A. aureolus			1														
	A. felis	7	15	1														0.002
	A. frankstonensis		1															
	A. pseudoviridinutans	1	1															
	A. udagawae	3	3															
	A. viridinutans		1															
	A. wyomingensis			1														
**OLO***	*A. arcoverdensis*					1												
	A. aureolus		1															
	A. felis		1	1	16	5												0.01
	A. frankstonensis				1													
	A. pseudoviridinutans				1	1												
	A. udagawae			1	5	1												
	A. viridinutans		1															
	A. wyomingensis			1														

Abbreviations: ITZ, itraconazole; VCZ, voriconazole; POS, posaconazole; ISA, isavuconazole; AMB, amphotericin B; MIF, micafungin; LUL, luliconazole; OLO, olorofim. *minimum inhibitory concentration values calculated; † minimum effective concentration calculated. Luliconazole data available for 16/17 AVSC environmental origin isolates (no data available for *A. udagawae* DTO 006-A3).

Supplementary Table 3. All AVSC *cyp51A* mutations found in this study compared to *A. fumigatus* wild-type *cyp51A*.

**Mutation present**	**Isolate ID/ Species**
P3S, V13G	6 *A. felis* isolates (DTO 341-E4, DTO 341-E5, DTO 341-E6, DTO 341-E8, DTO 341-E9, DTO 341-F1)
L5V	1 *A. wyomingensis* isolate (DTO 155-G2)
M11L, S493T (*S490T; ^S492T)	All 4 *A. pseudoviridinutans* and 32 *A. felis* isolates
A12V, L31H, T35A, Y137F, I151V, P376S, C409R, E488K, N512V	All 1 *A. siamensis* isolates (DTO 278-B6)
A18V	All 1 *A. viridinutans,* 2 *A. frankstonensis,* 9 *A. udagawae,* 3 *A. arcoverdensis,* 2 *A. aureolus,* 1 *A. siamensis* and 2 *A. wyomingensis* isolates
I19T, V44F, A177V	All 2 *A. frankstonensis* isolates
I19V, L316P	6 *A. felis* isolates (DTO 131-E3, DTO 327-G4, DTO 316-C8, DTO 159-C9, DTO 342-I6, DTO 342-I7)
F28L, D253G, M499V	1 *A. pseudoviridinutans* isolate (DTO 153-A1)
F29Y, P109A, N512E	All 3 *A. arcoverdensis* isolates
T35I, S117T, A330T	1 *A. udagawae* isolate (DTO 283-D3)
M39T, V480M	3 *A. felis* isolates (DTO 131-E4, DTO 131-E5, DTO 131-G1)
V44I, E317D, M383L, Q423H	All 9 *A.udagawae* isolates
T50A	All 4 *A. pseudoviridinutans* isolates
K59R	All 1 *A. viridinutans* and 2 *A. frankstonensis* isolates
A63S, L327P, V396A	All 1 *A. viridinutans* isolates

I71V	1 *A. arcoverdensis* isolate (DTO 019-F2)
V101L, A103T, A234V, I360V, V428I, G505R, Q423D, F478V	All 2 *A. aureolus* isolates
T215S, G138C	2 *A. felis* isolates (DTO 341-E4, DTO 341-E5)
D161N	All AVSC isolates (except 2 *A. udagawae* isolates [DTO 283-D3 and DTO 341-E3])
R171H	All 2 *A. frankstonensis* and 3 *A. arcoverdensis* isolates
R171K	6 *A. felis* isolates (CBS 143249, DTO 327-G4, DTO 316-C8, DTO 159-C9, DTO 342-I6, DTO 342-I7)
R171Q	All 1 *A. siamensis*, 2 *A. wyomingensis* and 31*A. felis* isolates (all *A. felis* except CBS 143249)
M172A	1 *A. udagawae* isolate (DTO 308-H6)
A189V	1 *A. pseudoviridinutans* isolate (DTO 342-I3)
S197C, Q340R	1 *A. felis* isolate (DTO 131-E6)
A234T, S241A	3 *A. pseudoviridinutans* isolates (DTO 303-A1, DTO 342-I3, DTO 304-I5)
K256N	All 2 *A. aureolus* and 1 *A. arcoverdensis* (DTO 019-F2) isolates
K256R, L390P, H474Y (^H473Y)	1 *A. pseudoviridinutans* isolate (DTO 304-I5)
D257E, K346R	All 1 *A. viridinutans,* 2 *A. frankstonensis* and 3 *A. arcoverdensis* isolates
N274S	19 *A. felis* isolates (DTO 327-G4, DTO 316-C8, DTO 159-C9, DTO 342-I6, DTO 342-I7, CBS 143249, DTO 131-E3, DTO 131-E9, DTO 131-G3, DTO 131-F4, DTO 131-F1, DTO 131-F2, DTO 131-F3, DTO 131-G2, DTO 155-G3, DTO 131-F9, DTO 131-F6, DTO 131-E6, DTO 155-G2)
K281H	All 4 *A. pseudoviridinutans* isolates
K281N, K314D	All 32 *A. felis* isolates
S302G	All 4 *A. pseudoviridinutans,* 9 *A. udagawae,* 1 *A. siamensis,* 2 *A. wyomingensis,* and 31 *A. felis* isolates (all *A. felis* isolates except CBS 143249)
K314E	All 1 *A. viridinutans,* 2 *A. frankstonensis,* 4 *A. pseudoviridinutans,* 9 *A. udagawae,* 3 *A. arcoverdensis,* 2 *A. aureolus,* 1 *A. siamensis* and 2 *A. wyomingensis* isolates
Q321R, L344I	1 *A. felis* isolate (DTO 341-F2)
N326S	10 *A. felis* isolates (DTO 131-E3, DTO 131-E9, DTO 131-G3, DTO 131-F4, CBS 143249, DTO 327-G4, DTO 316-C8, DTO 159-C9, DTO 342-I6, DTO 342-I7)
A330I	All AVSC isolates (except 1 *A. pseudoviridinutans* [DTO 303-A1] and 1 *A. udagawae* [DTO 283-D3])
K342E	8 *A. udagawae* isolates (DTO 006-A3, DTO 019-D7, DTO 019-D8, DTO 157-D8, DTO 166-D6, DTO 283-D3, DTO 308-H6, DTO 341-E3)

I354V(*I351V), M383V (*M380V)	All AVSC isolates (except all 9 *A. udagawae* isolates)
I360L (*I357L), V462L (*V459L; ^V461L)	All AVSC isolates (except all 2 *A. aureolus* isolates)
S373N (*S370N)	All 4 *A. pseudoviridinutans,* 2 *A. wyomingensis* and 31 *A. felis* isolates (all *A. felis* isolates except DTO 131-F6)
S373R	2 *A. arcoverdensis* isolates (DTO 316-F7 and DTO 316-F9)
L375F, C409Y, A419D	1 *A. udagawae* isolate (DTO 166-D6)
I384V	1 *A. udagawae* isolate DTO 341-E3, all 3 *A. arcoverdensis* isolates
P386S (*P383S), M499K (*M496K)	1 *A. pseudoviridinutans* isolate (DTO 303-A1)
N406S(*N403S; ^N405S)	All 4 *A. pseudoviridinutans,* 2 *A. wyomingensis* and 32 *A. felis* isolates
T420A (*T417A; ^T419A)	All 1 *A. viridinutans,* 4 *A. pseudoviridinutans,* 2 *A. aureolus,* 1 *A. siamensis,* 2 *A. wyomingensis,* and 32 *A. felis* isolates, and 3 *A. udagawae* isolates (DTO 166-D6, DTO 308-H6, DTO 341-E3)
T420S	5 *A. udagawae* isolates (DTO 157-D7, DTO 157-D8, DTO 019-D7, DTO 019-D8, DTO 283-D3)
T420V	All 2 *A. frankstonensis* and 3 *A. arcoverdensis* isolates
N425S	31 *A. felis* isolates (all *A. felis* isolates except DTO 341-E5)
V428F	17 *A. felis* isolates (DTO 131-E3, DTO 131-E6, DTO 131-E9, DTO 131-F1, DTO 131-F2, DTO 131-F4, DTO 131-F6, DTO 131-F9, DTO 131-G2 DTO 131-G3, DTO 155-G3, DTO 159-C9, DTO 316-C9, DTO 327-G4, DTO 342-I6, DTO 342-I7, CBS 143249)
V428L	1 *A. pseudoviridinutans* isolate (DTO 153-A1), 3 *A. udagawae* isolates (DTO 157-D7, DTO 019-D7, DTO 019-D8)
I471F	1 *A. aureolus* isolate (DTO 331-G6)
I471V (*I468V; I470V)	All AVC isolates (except 2 *A. arcoverdensis* [DTO 316-F7 and DTO 316-F9] and one *A. aureolus* [DTO 331-G6] isolates)
V480A	All 2 *A. wyomingensis* isolates
G482E	All 9 *A. udagawae* and 2 *A. wyomingensis* isolates
M499I	5 *A. felis* isolates (DTO 327-G4, DTO 316-C8, DTO 159-C9, DTO 342-I6, DTO 342-I7)
N512D (*N509D)	All AVSC isolates (except all 3 *A. arcoverdensis* and 1 *A. siamensis* isolates)
T513V	All 9 *A. udagawae*, 2 *A. aureolus* and 2 *A. wyomingensis* isolates
W6L, V15M, K80R, M172V[¥], D255G, C270S, I367L (*I364L), H403Y (*H400Y; ^H402Y), L464I (*L461I; ^L463I)	All 56 AVSC isolates

[¥]55/56 AVSC isolates tested had this mutation, and the remaining isolate (DTO 308-H6 [*A. udagawae*]) had a mutation at this position of M172A.

*,^ Due to difference in protein length to *A. fumigatus* wild-type *cyp51A*, the amino acid numbering for *A. pseudoviridinutans* isolates DTO 303-A1* and DTO 304-I5^ differ.

Supplementary Table 4. Verify3D results of the five *A. fumigatus* homology models (Model A-E) and three *Aspergillus viridinutans* species complex homology models (Model F-H).

Model	Species (isolate number)	% of residues that have scored a 3D-1D score >=0.2
A	*A. fumigatus* (AF338659)	83.11
B	*A. fumigatus* (AF338659)	84.66
C	*A. fumigatus* (AF338659)	84.47
D	*A. fumigatus* (AF338659)	84.08
E	*A. fumigatus* (AF338659)	84.65
F	*A. felis* (DTO 131-F6)	85.41
G	*A. viridinutans* (DTO 050-F1)	83.50
H	*A. aureolus* (DTO 278-B7)	82.91

Supplementary Table 5. Extrolite profiling for *Aspergillus viridinutans* species complex members.

Species	Extrolites produced

Aspergillus aureolus	antarone A<br>tryptoquivalines<br>tryptoquivalones
Aspergillus felis	5-N-acetyl-8-beta-isopropyl-ardeemin antafumicins and clavatol<br>cytochalasin E<br>cyclopiazonic acid<br>fumagillin<br>fumigatin<br>fumiquinazolines<br>fumitremorgin A & B<br>helvolic acid<br>kotanin<br>orlandin<br>pseurotin A1<br>rosellichalasin<br>tryptoquivalines<br>tryptoquivalones<br>verruculogen<br>viriditoxin<br>an aszonapyrone<br>an aszonalenin<br>an anthraquinone at RI 1208<br>many unique extrolites
Aspergillus frankstonensis	aszonapyrones<br>chrysogine-precursors<br>viriditoxin
Aspergillus pseudoviridinutans	antafumicins and clavatols<br>aspirochlorin<br>verruculogen<br>viriditoxin
Aspergillus udagawae	fumagillin<br>fumigatin<br>pseurotin A1<br>tryptoquivalines<br>tryptoquivalones
Aspergillus viridinutans	fischerin<br>fumitremorgin A & B

	kotanin<br>verruculogen<br>viriditoxin<br>an aszonalenin
Aspergillus wyomingensis	5-acetyl-8-beta-isopropylardeemin<br>fischerin<br>fumagillin<br>fumitremorgin A & B<br>helvolic acid<br>pseurotin A1<br>verruculogen

Supplementary Figures

Figure S1. Voriconazole (red), itraconazole (blue) and posaconazole (purple) binding to the haem group (grey) of *cyp51A* homology models F (*A. felis* [A]), G (*A. viridinutans* [B]), H (*A. aureolus* [C]), I and J (*A. felis* [D and E]).

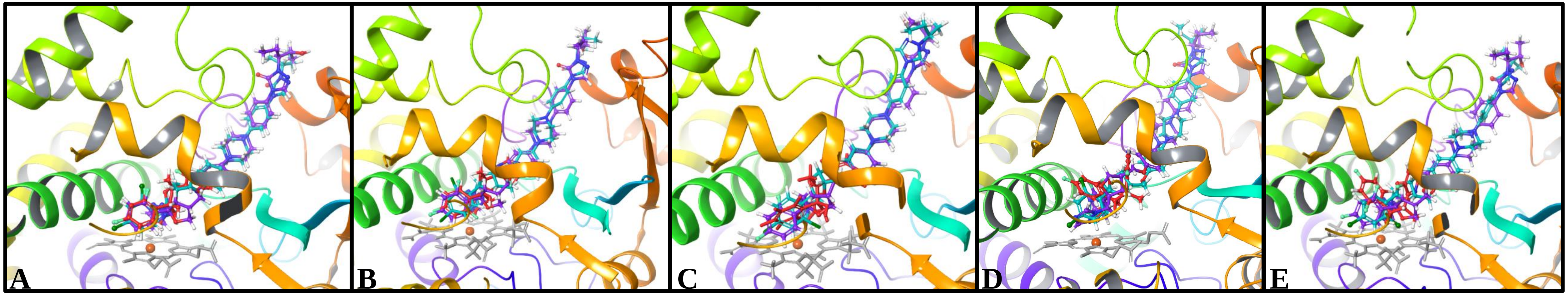
A
B
C
D
E
